# Assessment of Heat-Related Health Impacts in Brisbane, Australia: Comparison of Different Heatwave Definitions

**DOI:** 10.1371/journal.pone.0012155

**Published:** 2010-08-13

**Authors:** Shilu Tong, Xiao Yu Wang, Adrian Gerard Barnett

**Affiliations:** School of Public Health and Institute of Health and Biomedical Innovation, Queensland University of Technology, Brisbane, Queensland, Australia; Centre de Recherche Public de la Santé, Luxembourg

## Abstract

**Background:**

There is no global definition of a heatwave because local acclimatisation and adaptation influence the impact of extreme heat. Even at a local level there can be multiple heatwave definitions, based on varying temperature levels or time periods. We investigated the relationship between heatwaves and health outcomes using ten different heatwave definitions in Brisbane, Australia.

**Methodology/Principal Findings:**

We used daily data on climate, air pollution, and emergency hospital admissions in Brisbane between January 1996 and December 2005; and mortality between January 1996 and November 2004. Case-crossover analyses were used to assess the relationship between each of the ten heatwave definitions and health outcomes. During heatwaves there was a statistically significant increase in emergency hospital admissions for all ten definitions, with odds ratios ranging from 1.03 to 1.18. A statistically significant increase in the odds ratios of mortality was also found for eight definitions. The size of the heat-related impact varied between definitions.

**Conclusions/Significance:**

Even a small change in the heatwave definition had an appreciable effect on the estimated health impact. It is important to identify an appropriate definition of heatwave locally and to understand its health effects in order to develop appropriate public health intervention strategies to prevent and mitigate the impact of heatwaves.

## Introduction

Heatwaves can result in increased deaths and emergency hospital admissions, especially among vulnerable groups such as elderly people, young children and patients with chronic diseases. [1] Heatwaves are a significant but perhaps under-estimated environmental hazard as ambient thermal conditions have a significant impact on human health. [2]–[4] These impacts are highest during prolonged periods of unusual heat, such as the 15,000 deaths during the 2003 heatwave in France alone from the 1st to 20^th^ August. [5]

The magnitude of heatwave-related health effects depends on the intensity and duration of high temperatures, and also population acclimatisation and adaptation. [3], [5]–[7] What would be described as a heatwave in a temperate city may not be unusual in a tropical or subtropical city. These differences make it impossible to develop a global definition of a heatwave. [8] Even at a local level there can be disagreement about what constitutes a heat wave. For example, in Brisbane, the local health authority changed its definition in 2006. [9] This definition is used by a heat-warning system that gives advanced warning to the public and emergency services when forecast temperatures are above the heatwave threshold.

We are interested in the development of a heatwave definition and in whether changes in the definition are associated with appreciable changes in health outcomes. In this study, we compared heat-related health impacts using ten different definitions of heatwave in Brisbane, Australia.

## Materials and Methods

Brisbane is the capital city of Queensland, located in the south-east corner of the state, and has a sub-tropical climate with the latitude 27°29'S and longitude 153°8'E. It is Australia's third largest city (after Sydney and Melbourne), covering an urban area of 1326.8 km^2^ with a population of 992,176 on 30 June 2006. [10] In this study, we used daily time series data on emergency hospital admissions, air pollution and climatic variables collected for Brisbane city between January 1996 and December 2005. Mortality data from January 1996 to November 2004 (54,318 deaths totally) were also used. The mortality data were obtained up to November 2004 because of the delay in registering deaths by the government as all the data used in this study were acquired in 2006.

### Climate data

Information on climatic records from five monitoring stations in Brisbane city was provided by the Australia Bureau of Meteorology. The daily average values of minimum temperature, maximum temperature and relative humidity (RH) were computed using the records retrieved from these stations.

Maximum temperature was the highest measured in the 24 hours after 9 am in degrees Celsius. Minimum temperature was the lowest measured in the 24 hours before 9 am in degrees Celsius. Relative humidity is the amount of water in the air relative to the maximum amount of water that the air can hold at a given temperature (expressed as a percentage). Air temperatures and relative humidity were measured every three hours. The observations of air temperature and relative humidity at 3 pm were used to calculate the heat index. [11]–[13]

### Air pollution data

Air pollution data (including ambient 24-hour average concentrations of particulate matter with diameter less than 10 µm (PM_10_) and daily average maximum 1-hour nitrogen dioxide (NO_2_) and ozone (O_3_) concentrations) were provided by the Queensland Environmental Protection Agency. For each day, air pollution data were averaged from seventeen available monitoring stations across Brisbane.

### Emergency hospital admissions and mortality data

Daily data on emergency hospital admissions (EHAs) were provided by the Health Information Centre of Queensland Health. The data included principal diagnosis; the day, month and year of admission; and age group (years: 0–14, 15–64, 65–74, 75 +). Mortality data were provided by the Office of Economic and Statistical Research of the Queensland Treasury. The data included death of date, sex, age, and cause of death.

### Data analysis

Statistical analyses were undertaken using daily data on temperature, relative humidity, deaths and emergency hospital admissions. The heat index was calculated by a formula based on given air temperature and relative humidity. [11] Ten heatwave definitions (HWDs) were compared based on the local climate data (Table 1). The first three definitions have been widely used in the literature. [6], [11], [14] In order to more broadly assess the heat-related health impacts, we also used other seven definitions based on both the intensity and duration of heatwaves. For example, we assessed the health impacts of exposure to 35°C (i.e., top 1% of daily maximum temperature) for 2 or 3 consecutive days (HWDs 1 and 4), and also examined the health risk of exposure to 32.6°C (i.e., top 5% of daily maximum temperature) for up to 5 days (HWDs 8–10).

**Table 1:** Heatwave definitions (HWDs) and heatwave days during 1996–2005 in Brisbane, Australia.

**HWD**	**Definition**	**Heatwave days**	**Reference and note** a
1	The daily maximum temperature ≥35°C (about top 1%) for 3 or more consecutive days	6	Hansen et al. 2008^6^
2	The daily maximum temperature of more than 5 consecutive days exceeds the average maximum temperature by 5°C, the normal period being 1961–1990	10	Frich et al. 2002^13^
3	The heat index (maximum temperature + relative humidity) is expected to reach 40.6°C with a minimum temperature not below 26.7°C as a period of at least 48 h	3	Robinson 2001^10^
4	The daily maximum temperature would be equal to or greater than 35°C (about top 1%) for at least consecutive 2 days	20	Extended from HWD1
5	The daily maximum temperature would be equal to or greater than 37°C (about top 0.5%) for at least consecutive 2 days	7	Extended from HWD4
6	The top 2.5% (≥33.59°C) of daily maximum temperatures for a continuous 2 days period	49	Extended from HWDs4 & 5
7	The top 2.5% (≥33.59°C) of daily maximum temperatures for a continuous 3 days period	27	Extended from HWD6
8	The top 5% (≥32.65°C) of daily maximum temperatures for a continuous 3 days period	93	Extended from HWD6
9	The top 5% (≥32.65°C) of daily maximum temperatures for a continuous 4 days period	57	Extended from HWD6
10	The top 5% (≥32.65°C) of daily maximum temperatures for a continuous 5 days period	37	Extended from HWD6

a The first three definitions were widely used in the literature and the remainder (HWDs 4–10) were extended definitions developed for this study.

As a preliminary investigation the relationships between maximum temperature and mortality and emergency hospital admissions were explored using non-linear regression. We regressed the average daily number of deaths and EHAs against maximum temperature rounded to whole degrees C. We estimated the risk shape using a non-linear (quadratic) model, to capture the well known U-shaped association between temperature and health. [15]

Case-crossover analyses were used to assess the relationship between heatwaves and health outcomes using the ten HWDs. The case-crossover design is particularly useful here because it controls for trends and seasonal patterns in the dependent and independent variables. [16]–[18] We used the time-stratified case-crossover with a stratum length of 28 days, and additionally matched control days to case days using day of the week. The main independent variable was heatwave (categorised as yes/no). The dependent variable was either the daily number of deaths or the daily number of emergency hospital admissions. We also adjusted for the confounders including humidity and air pollution (ie, PM_10_, NO_2_ and O_3_).

## Results

Table 2 shows the daily summary statistics for maximum temperature, minimum temperature, relative humidity, mortality and emergency hospital admissions. Separate statistics are shown for the whole year and summer only in Brisbane between 1996 and 2005. The average daily deaths and EHAs varied from 5 to 43 and from 95 to 258, respectively. The maximum temperature was as high as 41.5°C with a mean maximum temperature of 26.3°C (standard deviation: 3.9°C); and the mean minimum temperature was 14.6°C (standard deviation: 5.3°C) for whole year.

**Table 2:** Summary statistics for daily temperature and health outcomes, 1996–2005 in Brisbane, Australia.

				**Percentile**					
**Variable**	**Mean**	**SD**	**Min**	**5**	**25**	**50**	**75**	**95**	**Max**
			**Whole year**						
Max temperature (°C)	26.3	3.9	12.6	20.2	23.3	26.4	29.2	32.7	41.5
Min temperature (°C)	14.6	5.3	0.4	5.2	10.7	15.4	18.8	22.3	27.6
Deaths	17	5	5	10	14	16	20	25	43
EHAs^a^	166	23	95	130	150	165	181	206	258
			**Summer**						
Max temperature (°C)	30.0	2.6	21.0	26.0	28.3	29.8	31.6	34.1	41.5
Min temperature (°C)	20.1	2.4	13.7	16.2	18.4	20.2	21.8	24.0	27.6
Deaths	16	4	5	9	13	15	18	22	43
EHAs^a^	161	24	95	124	145	160	175	201	258

a Emergency Hospital Admissions.

**Figure 1:**
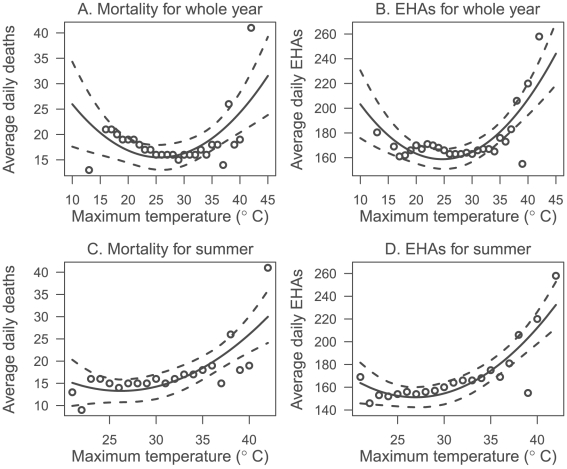
Scatter plots of the relationship between maximum temperature and mortality (A and C) and emergency hospital admissions (B and D) between 1996 and 2005. Fitted regression line (and 95% confidence interval) from a non-linear (quadratic) regression. EHAs: Emergency Hospital Admissions.

The average number (represented by the circles in the plot) of daily deaths and EHAs was clearly associated with maximum temperature for the whole year and just in summer between 1996 and 2005 in Brisbane (Figure 1). There is a U-shaped relation between maximum temperature and health outcomes as both the mean daily deaths and EHAs increased in winter and summer. The temperature threshold appears to be around 27°C (Figure 1 C and D).

Table 3 shows the estimated odds ratios (OR) of deaths and emergency hospital admissions during heatwave versus non-heatwave days. During heatwave days, there was a statistically significant increase in the odds ratios of emergency hospital admissions for all ten definitions. The odds ratios of mortality also increased for nine of the ten definitions using an unadjusted model, and eight of ten definitions using an adjusted model. Even a small change in the heatwave definition had an appreciable impact on the estimated morbidity and mortality. For example, the odds ratios of emergency hospital admissions varied from 1.03 to 1.18 for heatwave days compared to non-heatwave days (after adjustment for confounding factors). The results show that there is a strong association between heatwaves and health, but the strength of this association is variable and depends on the definition used.

**Table 3:** Odds ratios of emergency hospital admissions and mortality during heatwave periods using the case-crossover, 1996–2005 in Brisbane, Australia.

	Unadjusted		Partially Adjusted^b^		Fully Adjusted^c^	
HWD^a^	OR	95% CI^d^	OR	95% CI	OR	95% CI
**Emergency Hospital Admissions**						
HWD1	1.10	1.02, 1.18	1.10	1.02, 1.18	1.08	1.01, 1.16
HWD2	1.08	1.02, 1.14	1.07	1.01, 1.13	1.07	1.01, 1.13
HWD3	1.15	1.06, 1.26	1.13	1.03, 1.23	1.12	1.02, 1.22
HWD4	1.11	1.06, 1.15	1.09	1.05, 1.14	1.08	1.04,1.13
HWD5	1.20	1.13, 1.27	1.18	1.11, 1.25	1.18	1.11, 1.25
HWD6	1.06	1.04, 1.09	1.05	1.02, 1.07	1.04	1.01, 1.07
HWD7	1.10	1.06, 1.14	1.08	1.05, 1.12	1.08	1.04, 1.12
HWD8	1.06	1.04, 1.08	1.04	1.02, 1.06	1.04	1.02, 1.06
HWD9	1.06	1.03, 1.08	1.04	1.01, 1.07	1.03	1.01, 1.06
HWD10	1.08	1.05, 1.11	1.06	1.03, 1.09	1.06	1.03, 1.09
**Mortality**						
HWD1	0.89	0.71, 1.13	0.88	0.70, 1.12	0.87	0.68, 1.10
HWD2	1.01	0.85, 1.21	0.99	0.83, 1.18	0.99	0.83, 1.18
HWD3	1.82	1.39, 2.40	1.74	1.32, 2.29	1.73	1.32, 2.28
HWD4	1.31	1.15, 1.49	1.27	1.12, 1.45	1.26	1.10, 1.43
HWD5	1.60	1.33, 1.91	1.54	1.28, 1.85	1.53	1.27, 1.84
HWD6	1.21	1.11, 1.32	1.17	1.06, 1.28	1.16	1.06, 1.27
HWD 7	1.27	1.14, 1.41	1.22	1.09, 1.36	1.21	1.08, 1.35
HWD8	1.15	1.08, 1.23	1.11	1.04, 1.19	1.10	1.03, 1.18
HWD9	1.20	1.15, 1.33	1.15	1.06, 1.25	1.14	1.05, 1.24
HWD10	1.29	1.18, 1.41	1.24	1.13, 1.36	1.24	1.13, 1.36

a Heatwave definition;

b Adjusted for relative humidity and O_3_;

c Adjusted for relative humidity, PM_10_, NO_2_ and O_3_;

d CI: confidence interval.

## Discussion

There has been increasing research interest in assessing the health impact of heatwaves as the heat-related health risk is projected to rise in the coming decades because of climate change. [1] In this study, we found: i) small changes in the definition of heatwaves can lead to considerable differences in the risk assessment for heatwaves; ii) some commonly-used heatwave definitions do not appear to suit Brisbane (e.g., using HWDs 1 to 3); and iii) there was no conclusive evidence about which heatwave definition should be used, even though HWD 5 (i.e., the daily maximum temperature greater than or equal to 37°C—approximately the top 0.5% of temperatures—for at least 2 consecutive days) seems to be superior than other HWDs when the impact of heatwaves on both mortality and EHAs is considered. The intense of heat in HWD 5 seems to be greater than most definitions in Table 1. Figure 1 showed that the temperature threshold at which deaths and emergency hospital admissions began to rise was around 27°C in Brisbane, which is higher than the thresholds observed in most cities from Europe and the United States. [2], [15], [19] A high threshold for Brisbane appears reasonable because it is a subtropical city with a population acclimatised to heat.

Different definitions for heatwave have been used in recent publications. For example, Anderson and Bell reported that, comparing the 99th and 90th percentile temperatures for the community in the United States, heat-related mortality was most associated with a shorter lag (average of same day and previous day), with an average increase of 3.0% in mortality risk (95% posterior interval: 2.4% to 3.6%). [2] The study also found that heat effects were generally larger in the northern USA compared with the south, probably because people in the south are better adapted to hot weather. Hansen et al. defined heatwaves in Adelaide, Australia as three or more consecutive days when the daily maximum temperature reached or exceeded 35°C. [6] Compared with non-heatwave periods, hospital admissions increased by 7.3% during heatwaves. In another study, Knowlton and colleagues used a variety of locally varying definitions of “heatwave” in assessing the impact of the 2006 California heatwave on hospitalisations and emergency department visits. [20] They suggested that a heatwave definition based on a higher temperature threshold would be associated with a greater increased relative risks for hospital admissions compared with emergency department visits. Metzger et al. examined a range of different temperature measures and found that the maximum heat index over the previous three days gave the best estimates of heat-related mortality in New York. Evidently, there is a need to evaluate locally valid HWDs and their impact on health outcomes. [21]

This study has three major strengths: 1) this is the first study to broadly examine heat-related health effects using different kinds of HWDs. The results of the study demonstrate that it is fundamentally important to evaluate how a heatwave is defined when a comparison of heat-related health effects is made; 2) the data used in this study were of good quality, with no missing values; and 3) importantly, we were able to adjust for the possible confounding effects of air pollution and humidity.

This study also has two key weaknesses. Firstly, it focused on only one city. However, the finding that different heatwave definitions lead to variations in the size of the health outcomes is likely to apply to other locations. Secondly, we only considered the effect of heatwaves on total mortality and emergency hospital admissions, but did not examine different causes of deaths or specific types of admissions. This issue will be addressed in further research.

Finding the best definition of a heatwave is a challenging research question. [3], [22] This study used ten different heatwave definitions, including both absolute and relative ones, and demonstrated that even a small change in the definition had a considerable impact on the estimated risk of heatwave. As global warming continues, the frequency, intensity and duration of heatwaves are likely to increase. [1] Thus, a community-based response plan for heatwave is necessary to ensure that prompt and appropriate public health intervention can be implemented to minimise the impact of heatwaves. It is important to identify an appropriate definition of heatwave locally and to understand its local health effects in order to develop appropriate public health intervention strategies to prevent and mitigate the impact of heatwaves. It is also one of the fundamental research issues which need to be addressed when we compare the heat-related health impacts across different regions.
